# HORI: a web server to compute Higher Order Residue Interactions in protein structures

**DOI:** 10.1186/1471-2105-11-S1-S24

**Published:** 2010-01-18

**Authors:** Pandurangan Sundaramurthy, Khader Shameer, Raashi Sreenivasan, Sunita Gakkhar, Ramanathan Sowdhamini

**Affiliations:** 1 National Centre for Biological Sciences (TIFR), GKVK Campus, Bellary Road, Bangalore, 560065, India; 2 Department of Mathematics, Indian Institute of Technology Roorkee, Roorkee 247667, Uttarakhand, India; 3 Centre for Biotechnology, Anna University, Chennai 600025, India; 4 Department of Bioinformatics, School of Biotechnology, Karunya University, Coimbatore - 641114, India; 5 University of Wisconsin-Madison, Madison, WI 53706-1481, USA

## Abstract

**Background:**

Folding of a protein into its three dimensional structure is influenced by both local and global interactions within a protein. Higher order residue interactions, like pairwise, triplet and quadruplet ones, play a vital role in attaining the stable conformation of the protein structure. It is generally agreed that higher order interactions make significant contribution to the potential energy landscape of folded proteins and therefore it is important to identify them to estimate their contributions to overall stability of a protein structure.

**Results:**

We developed HORI [Higher order residue interactions in proteins], a web server for the calculation of global and local higher order interactions in protein structures. The basic algorithm of HORI is designed based on the classical concept of four-body nearest-neighbour propensities of amino-acid residues. It has been proved that higher order residue interactions up to the level of quadruple interactions plays a major role in the three-dimensional structure of proteins and is an important feature that can be used in protein structure analysis.

**Conclusion:**

HORI server will be a useful resource for the structural bioinformatics community to perform analysis on protein structures based on higher order residue interactions. HORI server is a highly interactive web server designed in three modules that enables the user to analyse higher order residue interactions in protein structures. HORI server is available from the URL: http://caps.ncbs.res.in/hori

## Background

Derivation of a three-dimensional structure of a protein from its primary sequence is controlled by a complex and largely unknown set of principles called as folding code. Folding principles of a three-dimensional structure is largely under the influence of both global and local interactions. For example, pairwise, triplet and quadruple based higher order residue interactions play a crucial role to attain the stable conformation of the protein structures. Higher order residue interactions also contribute to the potential energy landscape of proteins and hence it is important to understand such interactions mediated in the level of active site residues to whole structure [1-7]. In the current era of high-throughput sequencing, due to huge lacunae in the sequence to structure ratio, computational approaches are playing a significant role in understanding the design principles and functional aspects of protein structures [8-15]. In this paper, we describe the availability of a web server called HORI (Higher Order Residue Interactions in proteins) developed for the calculation of generic and specific higher order residue interaction patterns in protein structure. The basic algorithm of HORI is designed based on the classical concept of four-body nearest-neighbour propensities of amino acid residues. It has been proved that higher order residue interactions, up to the level of quadruple interactions, will play a major role in the three-dimensional structure of proteins. According to the earlier studies, if we approximate each residue as a sphere centred on its location, it is possible for a maximum of four closely packed spheres to make mutual contact, thus giving rise to pair wise, triplet and quadruple interactions. Just as no more than four same-sized spheres can be in mutual contact in 3D space, higher order interaction beyond quadruple interactions are generally not considered [3,16]. The concept of higher order interactions has been introduced and successfully employed in structure analysis and fold recognition by different groups [17,18]. Earlier work also reported that the higher order interactions can be used to improve accuracy of fold recognition and generic structure analysis [6,19]. As HORI server can be used to compute higher order interactions in different levels from single residue to whole structure, analysis of higher order interactions mediated by residues in the functional or active sights will provide better insights to understand the structural interactions contributed by these important residues. We envisage that availability of a server to compute higher order interactions will enable the users to perform the computation of higher order interactions in easy steps. In this manuscript, we explain various feature of HORI server along with different example scenarios where the general application of the higher order interaction and the server is useful.

## Methods

### HORI server implementation: description and features

**Figure 1:**
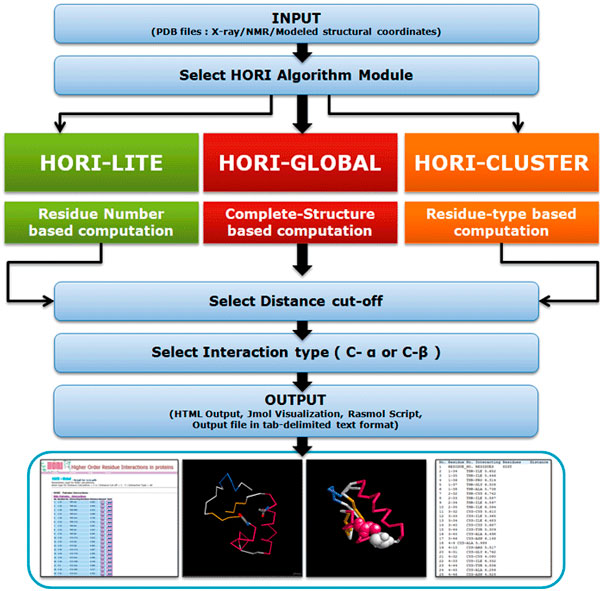
**HORI Server flow chart**.

**Figure 2:**
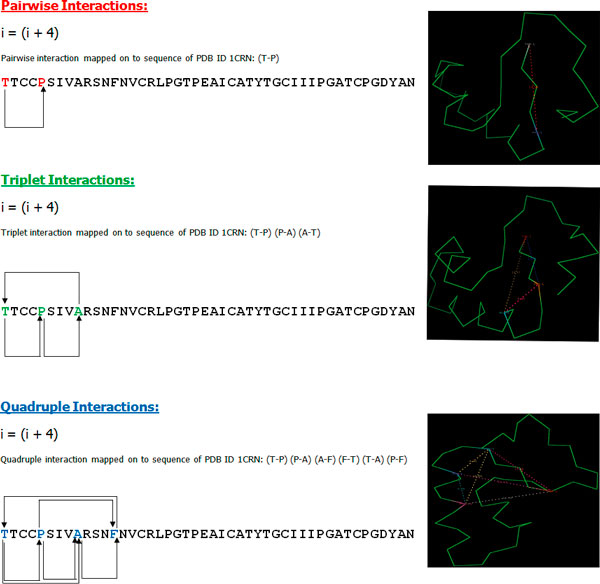
**Different Higher order residue interactions mapped on to Crambin structure (PDB ID: **1CRN).

A detailed flow chart of different modules available from HORI server is provided in Figure 1. HORI Server is designed as three distinct modules: HORI - Global, HORI - Lite, and HORI - Cluster. Three different modules are provided with different options to compute generic and specific interactions from protein structures. All the programs in the three different modules are available for the computation based on C^α ^and C^β ^atom types. Different web interfaces are available for both single chain and multi-chain based computations. HORI Server provides user-friendly, interactive interfaces for the submission of files and visualization of results. Short description and graphical representation about the approach used for the calculation of pairwise, triplet and quadruple interactions are explained in Figure 2. Web interface of HORI server is developed using HTML and JavaScript. HORI programs are coded in C-language and compiled using GNU Compiler (gcc version 4.2). Interactive visualization of higher-order interactions on query structure is implemented using Jmol[20]. Rasmol [21] scripts are also provided for the interactive analysis of structures in local machine. Wrapper scripts and automated e-mail programs are coded in Perl. HORI server is running on an Apache web server powered by Athlon Quad-core processors.

### HORI - Global

HORI - Global provides set of programs for the computation of higher order interactions at the complete structural level. Using options in HORI - Global module, user can compute pairwise, triplet and quadruple interactions among the residues in a given protein structure. Here, the complete set of all possible interactions in each category will be computed. The higher and lower-cut distance cut-off for identifying probable higher order interactions are provided as a user-defined option. Preferred range for higher order residue interactions are 1 - 7 Angstroms. Using efficient utilization of different parameters, like atom-type for distance calculation, interaction type and distance cut-off, and user can derive interesting information on higher order interactions from the protein structure of interest. HORI - Global is the computationally intensive module available in the HORI server. This module is designed as an email based server due to computation intensive nature of the programs.

### HORI - Lite & HORI - Cluster

Hori - Lite, the second module available from HORI server offers a set of programs for the computation of higher order interactions in a structure, based on specific residue numbers and specific distance. The third module, HORI - Cluster, offers a set of programs for computation of higher order interactions of different types of residues in a structure. Both HORI - Lite and HORI - Cluster provide nine different programs under the category of three different classes of higher order interactions. Programs are available in pairwise interactions class to compute pairwise distances for any two residues in PDB file and pairwise distance around any one residue. Triplet interaction class provides programs to compute triplet distance for any three residues, triplet distance for around any two residues and triplet distance around any one residue. Quadruplet interaction class provides options to compute quadruple Distance for any four, three, two or one residue in a PDB file.

### Input options

All the three modules of HORI server require three dimensional co-ordinates of protein structures in PDB format. User can submit structures from PDB files and modelled structure files in PDB format. User can supply chain of interest from the various NMR structures. In the current version, HORI server can be used to analyse both single chain and multi-chain proteins. Due to computational-intensive nature of HORI-Global programs, currently, the server allows only two chains in the multi-chain based higher order interaction calculations. User of HORI - Global module should also submit a valid, non-commercial email address to the server to receive the notification about the availability of results. HORI server will send the result URL to the email address. In comparison to HORI - Global, both HORI - Lite and HORI - Clusters offer specific and faster computation of the higher order interactions in a protein structure. General parameters in different modules of HORI server are atom type, interaction type and range of distance to calculate interactions. Apart from these general parameters, user can also mention the range of residues (option available in HORI - Global), exact residue numbers (available as a parameter in HORI - Lite) and amino acid type (available as a parameter in HORI - Cluster).

### Output details

HORI server computes pairwise, triplet and quadruple interactions within a protein structure based on different parameters provided by the user. Among the three modules available in HORI server, HORI - Global provides more detailed output. User can generate customised output based on pairwise, triplet and quadruple computations using HORI - Global. HORI - Global module also provides a tab-delimited text file of the results for the customised analysis of higher order interactions by the user. User can also visualize the interaction, either using pre-installed Rasmol [21] on local machines or on browser using Jmol plug-in [21]. All three modules in HORI server provides output in html, tab-delimited text files for parsing and further analysis of higher-order interactions and visualization options to see individual interactions.

## Results and discussion

Bioinformatics tools are widely used in the study of protein structures to understand structural, functional and interaction aspects of protein structures. Several tools are also available for the calculation of interaction, interface, bonding patterns, disulphide connectivity. In PDBWiki [22] various tools are listed to define or select interacting residues. For example, Protein Interaction Calculator [23] can be used to calculate several interaction parameters like intra-protein interactions, solvent accessibility, protein-protein interactions and depth calculations. Other tools like SCOPPI [24] can be used for analysis of protein-protein interface, LPC/CSU [25,26] can be used for ligand-protein contacts & contacts of structural units. Irrespective of such wide array of structure tools for protein structure analysis, according to the best of our knowledge, HORI server is a primary attempt to provide a web server for the computation of higher order residue interactions in proteins in a whole structure as well residue-specific level.

### Applications of HORI Server in protein structure analysis

Higher order interactions calculated using set of computationally intensive algorithms available in HORI server will be useful in fold prediction, protein modelling, protein-protein interaction, active site identification and to understand higher order interaction characteristics of active site residues within specified distance shells. Knowledge about the higher order interactions will be of great importance in structural biology due to its wide range of applications in fold recognition, structural analysis, protein engineering, protein-protein interactions, active site identification and to understand mechanism of action of enzymes [6,17,18]. In order to illustrate the usefulness of higher order interactions, we enumerate four different examples, in protein structure analysis contexts, where HORI server is used to analyse set of different single chain and multi-chain crystal structures from PDB.

### Analysis of Higher order interactions in structures from TIM fold and Rossmann fold

**Figure 3:**
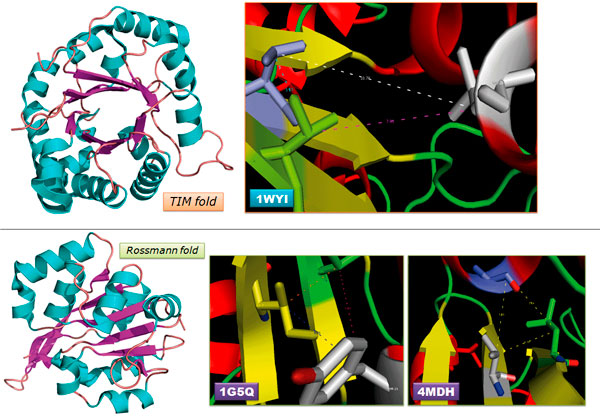
**Triplet interactions in representative members of TIM fold and Rossmann fold**.

Prior knowledge of spatial and higher order interactions will add more value to the interpretation of HORI results and to discriminate between folds of roughly similar secondary structural topology but dissimilar overall shape. To demonstrate where triplet and quadruplet interactions might be crucial, we take the example of two α/β folds from SCOP database [27,28] with strikingly similar secondary structural topology but distinct overall spatial arrangements: triose phosphate isomerase (TIM) fold [29] and the doubly-wound dehydrogenase or Rossmann-fold [30]. Despite the similar repeats of super secondary structural elements (βαβ), the TIM barrel is a closed structure with the first and eighth β-strand in hydrogen bonding, whereas the doubly-wound Rossmann folds have open structures with two distinct domains holding the entire polypeptide fold. Due to the similar secondary structural arrangements, most fold recognition methods have failed to distinguish the correct fold. When the pairwise interactions between the residues in the first two β strands in 1WYI (TIM fold) [31] and 1G5Q (Rossman fold) [32] are compared, they lie in the same range. The pairwise interaction between the C^β ^atoms of residues 8 and 39 that lie in adjacent parallel strands in 1WYI (TIM fold) is found to be 5.17 Å while that between residues 7 and 34 in 1G5Q (Rossman fold) is 4.49 Å. While the topology appears to be similar, the higher order interactions exhibit distinct profiles for the two proteins. While the triplet interactions between C^β ^atoms of residues 7, 21, 34 in 1G5Q lie within 10 Å, it is not so in the case of the triplet formed between C_α _atoms of residues 8, 28, 39 in 1WYI. In the case of 4MDH[33], which is a Rossman fold protein, there are no triplet interactions observed between adjacent β strands and the joining α helix. For example, the only triplet interaction observed for C_α _atom of residue 8 is with residue 38 of another β strand and residue 76 of α helix. On the other hand, in 1WYI, residue 8 is seen forming triplet interactions with adjacent β strands on either side, forming the triplet residues-8, 231, 244 and residues-8, 28, 39. Graphical view of the triplet interactions in 1WYI, 1G5Q and 4MDH is provided in Figure 3. HORI server can be used in the analysis of distance based interactions within proteins and proves to be an effective means of distinguishing protein folds through its map of higher order interactions.

### Identification of intramolecular higher order interaction mediated by a cysteine residue in Crambin

**Figure 4:**
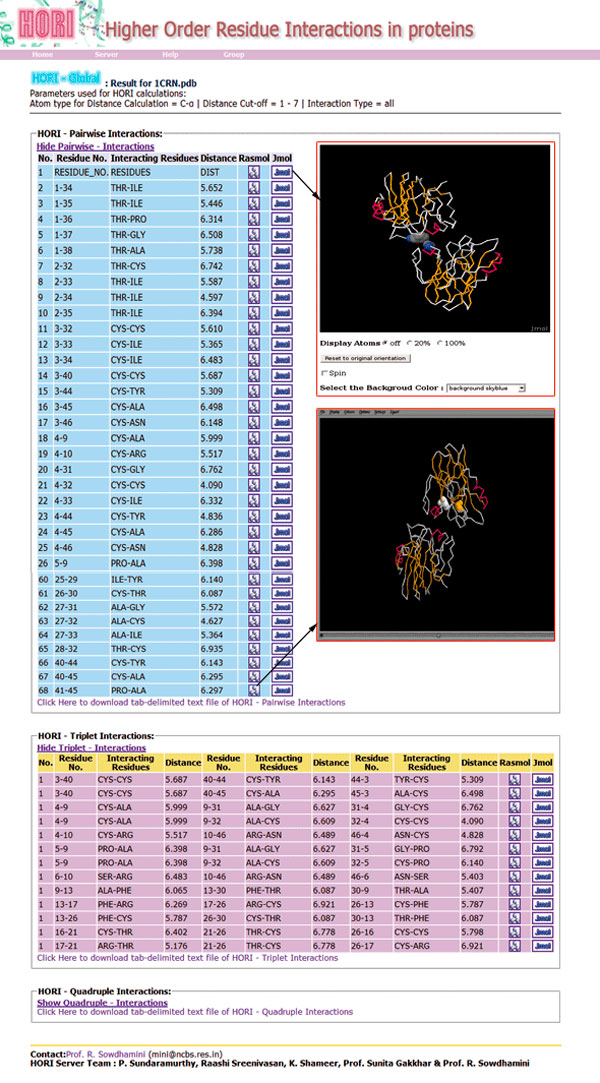
**Detailed screenshot of HORI server with results of HORI-Global runs on crambin (PDB ID - **1CRN).

Crambin [34] is a member of Crambin-like superfamily plant seed protein with three disulphide bonds. We have used HORI Server to compute quadruple distances around 1 cysteine residue in Crambin structure (PDB ID: 1CRN) with in the distance range of 1-7 Angstroms. This option will provide an insight in to all intramolecular quadruple interactions mediated by the six cysteine residues in crambin [35]. A detailed screenshot of HORI server with results of HORI-Global runs on crambin (PDB ID: 1CRN) is provided in Figure 4.

### Identification of alternate active site residues and suitability of residues for mutational studies based on higher order residue interaction

Cutinase [36] is a serine esterase containing the classical Ser, His, Asp triad of serine hydrolases [37]. Catalytic Site Atlas [38] reports five active site residues based on homologous entries, found by PSI-BLAST [39] alignment to one of the PDB entries (PDB ID: 1AGY). We used two of these residues to identify potential third interacting residue (using the compute triplet distances option) around any two residues in PDB file (PDB ID: 1CEX). Approaches like this can be useful to identify potential alternate active site residues and residues suitable for mutational studies, based on number of intermolecular interactions contributed by residues in active site regions.

### Analysis of higher order interaction in a multi-chain protein involved in 3D domain swapping

CD2 is shown to have ability to fold in two ways as a monomer or as a swapped dimer [40,41]. We have performed HORI-Global based computation of higher order residue interactions using the two chains of CD2 structure (A and B chains of PDB ID: 1A64). The higher order interactions within the cut-off of 0-8 Å clearly indicate that the swapped structure is stabilised by several higher order interactions between the residues in chains A and B [42].

## Conclusion

HORI server provides a landscape of all possible higher order residue interactions in protein structures. The information provided by HORI server will be important to understand the role of higher order residue interaction in stability, to recognise alternate patches of functionally important residues, structural integrity and folding properties of modelled and experimentally solved protein structures. Availability of HORI server in the public domain will enable the structural bioinformatics community to analyze and study higher order interaction patterns from protein structure data in easier way and gain better insight about the structure. This can also aid the design of mutation experiments for biochemists and biologists. By providing various options in three different modules, HORI server offers a complete computing platform online for higher order residue interactions and for the analysis of protein structures.

## Competing interests

The authors declare that they have no competing interests.

## Authors' contributions

S.G and R.S. designed the research components and applications of this project. The project was conceived and developed by P.S. R. Sreenivasan coded the core calculations. K. S. contributed to the coding, developed and compiled the scripts for automation and generation of the webserver. P. S, K. S and R. Sreenivasan wrote the first draft of the manuscript. S. G and R. S provided critical inputs to improve the manuscript.
